# Crystal structure and Hirshfeld surface analysis of a supra­molecular aggregate of 4-formyl-*N*,*N*-di­methyl­anilinium bromide with tetra­bromomethane

**DOI:** 10.1107/S2056989025006929

**Published:** 2025-08-07

**Authors:** Atash V. Gurbanov, Tuncer Hökelek, Gunay Z. Mammadova, Khudayar I. Hasanov, Tahir A. Javadzade, Alebel N. Belay

**Affiliations:** a Excellence Center, Baku State University, Z. Khalilov Str. 23, AZ 1148 Baku, Azerbaijan; b Hacettepe University, Department of Physics, 06800 Beytepe-Ankara, Türkiye; c Department of Chemistry, Baku State University, Z. Khalilov Str. 23, AZ 1148 Baku, Azerbaijan; d Azerbaijan Medical University, Scientific Research Centre (SRC), A. Kasumzade Str. 14, AZ 1022 Baku, Azerbaijan; e Department of Chemistry and Chemical Engineering, Khazar University, Mahsati Str. 41, AZ 1096 Baku, Azerbaijan; f Department of Chemistry, Bahir Dar University, PO Box 79, Bahir Dar, Ethiopia; University of Kentucky, USA

**Keywords:** crystal structure, non-covalent inter­actions, hydrogen bond, benzenaminium

## Abstract

The title compound, C_9_H_12_NO^+^·Br^−^·CBr_4_, consists of one 4-formyl-*N*,*N*-di­methyl­benzenaminium bromide and a tetra­bromo­methane mol­ecule. In the crystal, the bromide ions link 4-formyl-*N*,*N*-di­methyl­benzenaminium moieties through inter­molecular C—H⋯Br and N—H⋯Br hydrogen bonds, while inter­molecular C—H⋯O hydrogen bonds link 4-formyl-*N*,*N*-di­methyl­benzenaminium cations, enclosing *R*^2^_2_(18) ring motifs, into a di-periodic network structure. The tetra­bromo­methane mol­ecules fill the spaces between the layers. Neither π–π nor C—H⋯π(ring) inter­actions are observed. A Hirshfeld surface analysis of the crystal structure indicates that the most abundant contacts contributing to the crystal packing are from H⋯Br/Br⋯H (56.0%), Br⋯Br (12.1%), H⋯O/O⋯H (9.7%) and H⋯H (9.5%) inter­actions.

## Abstract 2

In the title compound, bromide ions link 4-formyl-*N*,*N*-di­methyl­benzenaminium mol­ecules through inter­molecular C—H⋯Br and N—H⋯Br hydrogen bonds, while inter­molecular C—H⋯O hydrogen bonds link the cations, enclosing *R*^2^_2_(18) ring motifs, into a di-periodic network structure. The tetra­bromo­methane mol­ecules fill the spaces between the layers.

## 1. Chemical context

Aldehydes are versatile compounds for the synthesis of organic acids, dyes, drugs, perfumes, detergents, soaps, *etc*. In the synthesis of those compounds the aldehydes undergo many different nucleophilic addition reactions. In order to increase the electrophilicity of the carbon atom at the C=O group of the aldehyde mol­ecule, metal complexes or organocatalysts are commonly used (Ma *et al.*, 2017 ▸, 2021 ▸; Mahmudov & Pombeiro, 2023 ▸). Following crystal engineering principles (Gurbanov *et al.*, 2020 ▸; Mahmoudi *et al.*, 2018 ▸; Velásquez *et al.*, 2019 ▸), weak inter­actions, halogen bonds, and other inter­actions, have been used in the activation of aldehydes towards the synthesis of various classes of organic compounds (Gurbanov *et al.*, 2022 ▸; Sutar & Huber, 2019 ▸). We found that weak inter­actions can be formed with substituents at the aldehyde mol­ecules instead of with the oxygen atom of the C=O group.
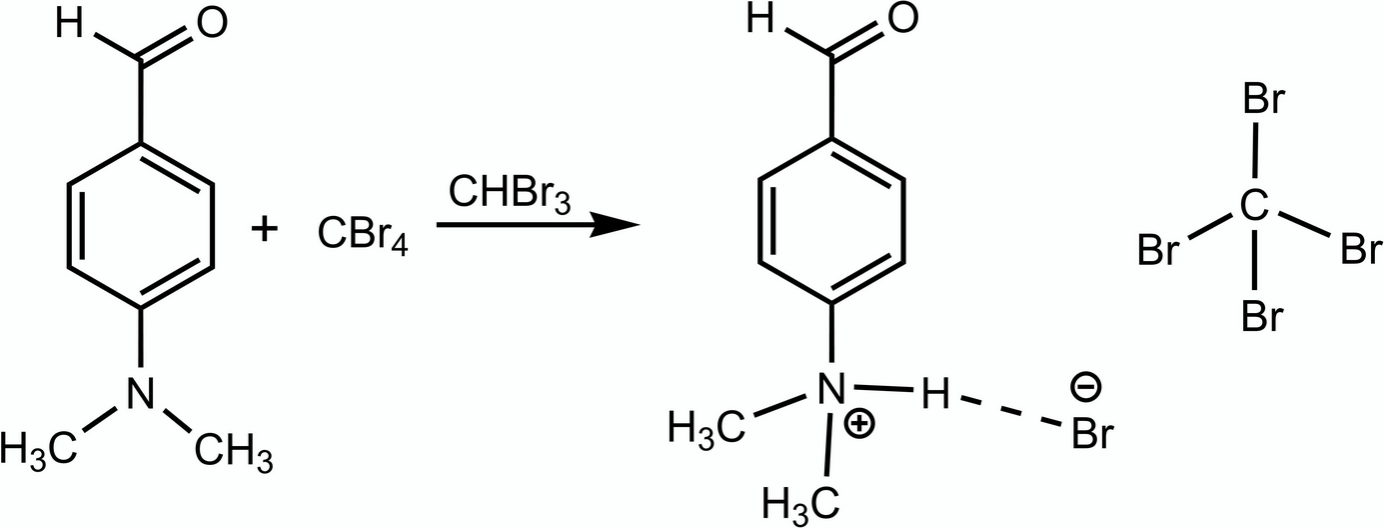


Herein, we provide details of the synthesis and an examination of the mol­ecular and crystal structures, together with a Hirshfeld surface analysis, of the title compound (I).

## 2. Structural commentary

**Figure 1:**
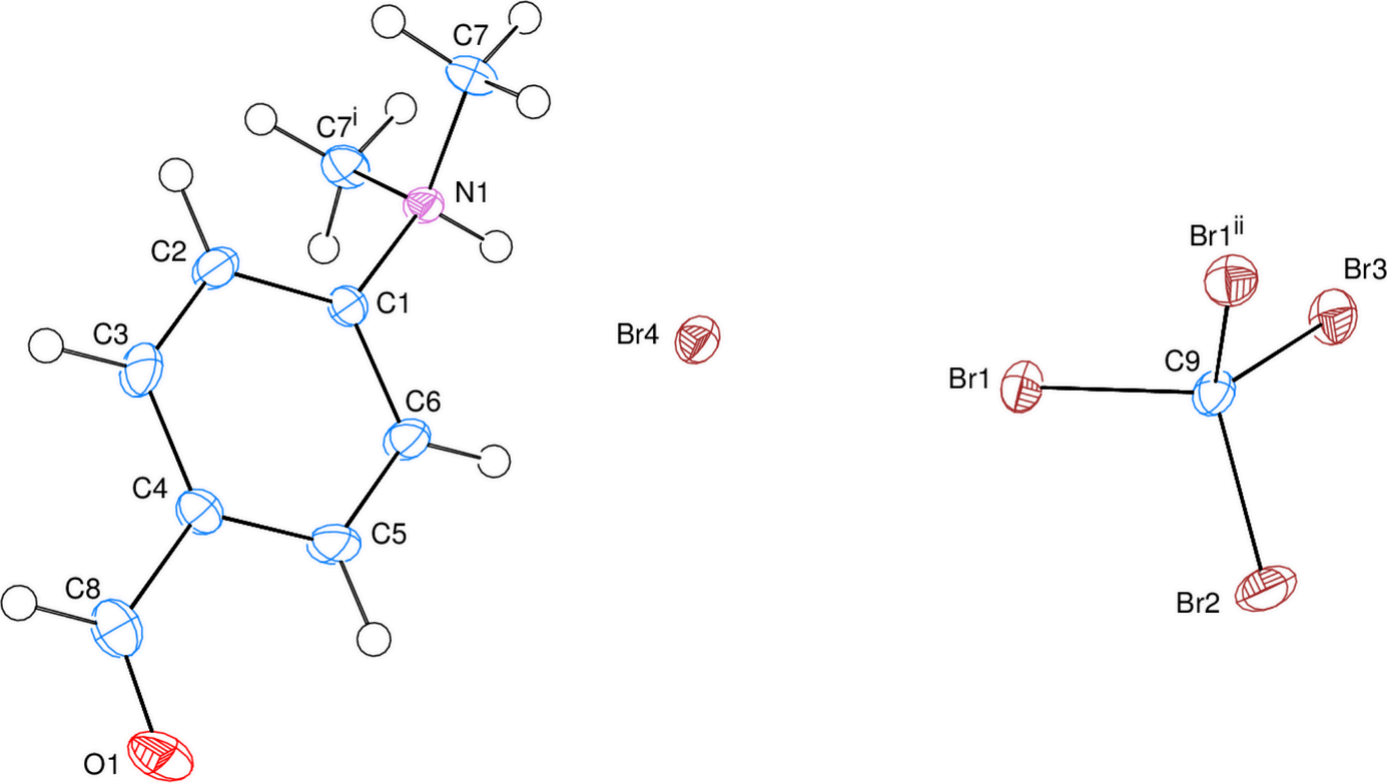
The title compound with atom-numbering scheme and 50% probability ellipsoids. Symmetry codes: (i) *x*, −*y* + 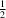, *z*; (ii) *x*, −*y* + 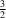, *z*.

The title compound, (I), consists of one 4-formyl-*N*,*N*-di­methyl­benzenaminium bromide unit and a tetra­bromo­methane solvent mol­ecule (Fig. 1 ▸). The C—C and C—C—C bond lengths and angles of ring *A* are in the ranges 1.366 (7) to 1.3998 (10) Å and 118.6 (4) to 121.8 (4)° with average values of 1.388 (8) Å and 120.0 (4)°, respectively. These values are reported as 1.375 Å and 119.9° in *p*-di­methyl­amino-benzaldehyde hydro­bromide, (II) (Dattagupta & Saha, 1973 ▸). Both of the N—C bonds between the methyl carbon and amino nitro­gen atoms are 1.497 (4) Å, and the corresponding ones in compound (II) are 1.51 (4) and 1.43 (4) Å. The C=O bond length in the aldehyde group is 1.198 (7) Å, and its corresponding value is 1.18 (4) Å in compound (II). The dihedral angle between ring *A* and the plane of atoms (O1/C4/C8) is 0.00 (2)° while the corresponding value in compound (II) is 1.39°. The C4—C8 [1.467 (7) Å] bond length is in good agreement with the theoretically calculated single-bond lengths between trigonally linked (*sp*^2^) carbon atoms: 1.479 Å (Dewar & Schmeising, 1959 ▸) and 1.477 Å (Cruickshank & Sparks, 1960 ▸). The corresponding exocyclic C—C bond length is reported as 1.38 (4) Å in compound (II).

## 3. Supra­molecular features

**Figure 2:**
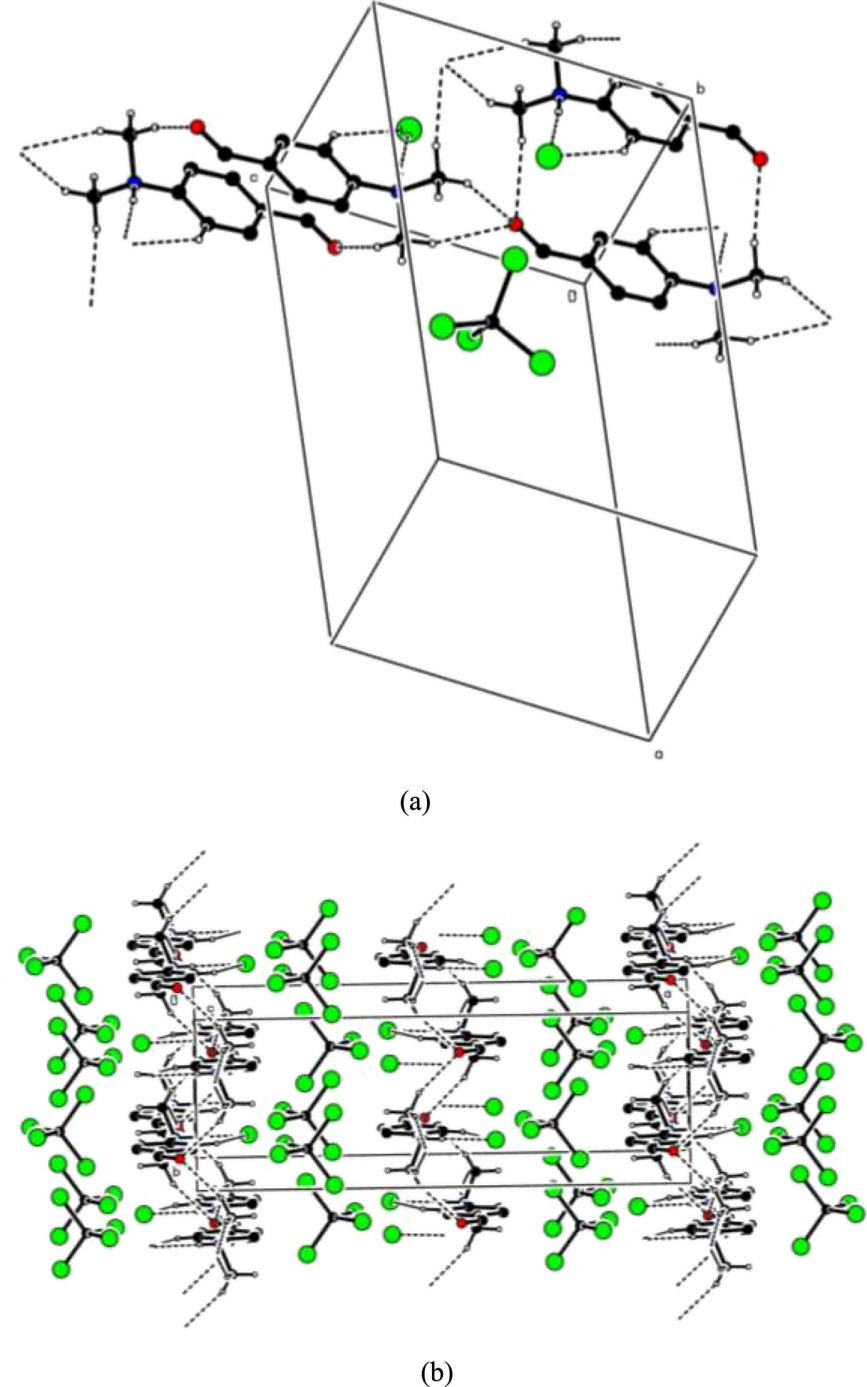
(*a*) A partial packing diagram showing the presence of an 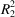
(18) ring motif (upper right). (*b*) A packing diagram viewed approximately down the *c*-axis direction. Inter­molecular C—H⋯Br, N—H⋯Br and C—H⋯O hydrogen bonds are shown as dashed lines. Hydrogen atoms not involved in hydrogen bonds have been omitted for clarity.

In the crystal, inter­molecular C—H⋯Br and N—H⋯Br hydrogen bonds link the bromide ions and the 4-formyl-*N*,*N*-di­methyl­benzenaminium moieties (Table 1 ▸ and Fig. 2 ▸*a*). At the same time, inter­molecular C—H⋯O hydrogen bonds (Table 1 ▸) link pairs of mol­ecules through 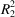
(18) hydrogen-bonding motifs, into a di-periodic network structure (Fig. 2 ▸*a*). The tetra­bromo­methane solvent mol­ecules occupy the spaces between the layers (Fig. 2 ▸*b*).

## 4. Hirshfeld surface analysis

**Figure 3:**
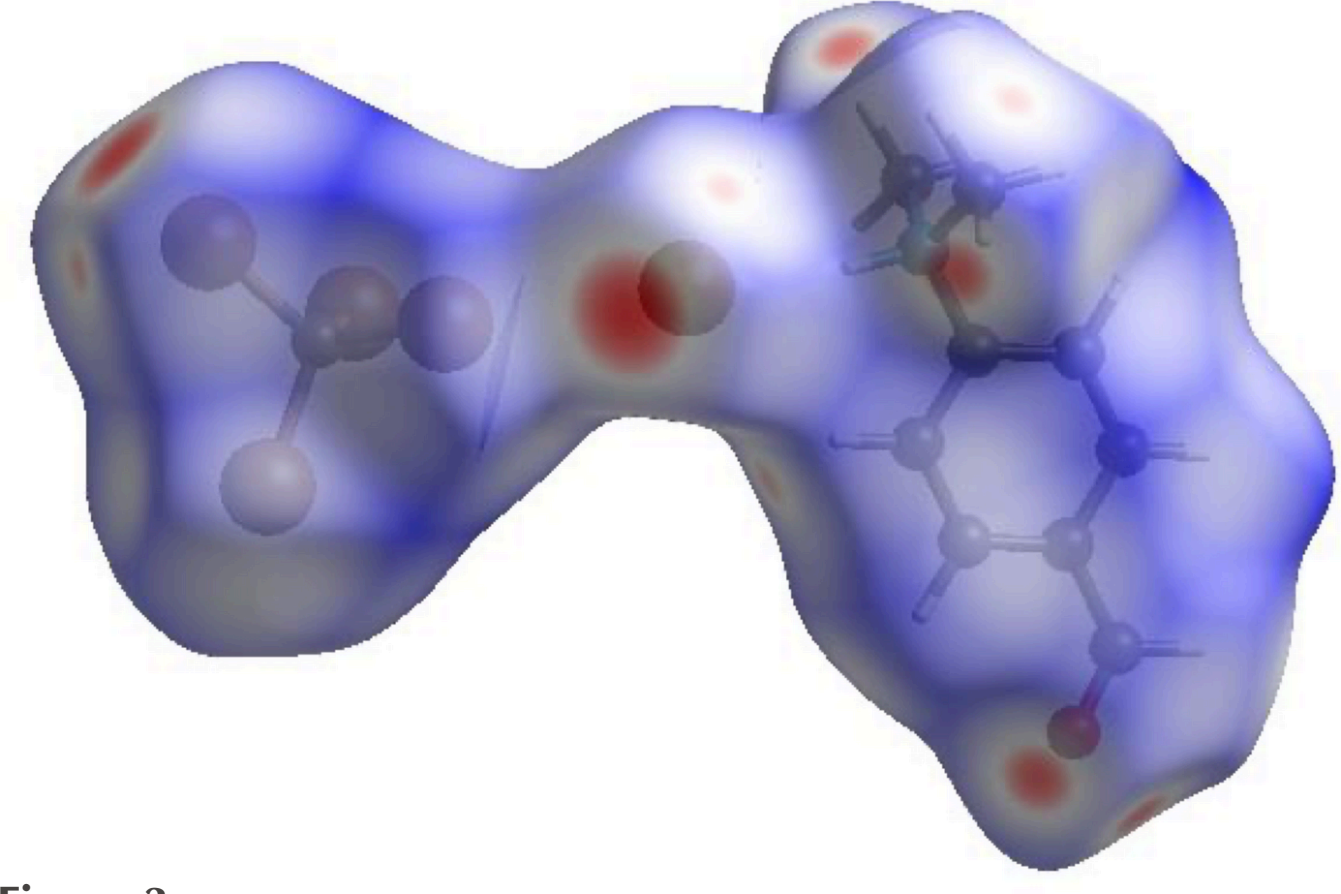
View of the three-dimensional Hirshfeld surface plotted over *d*_norm_.

**Figure 4:**
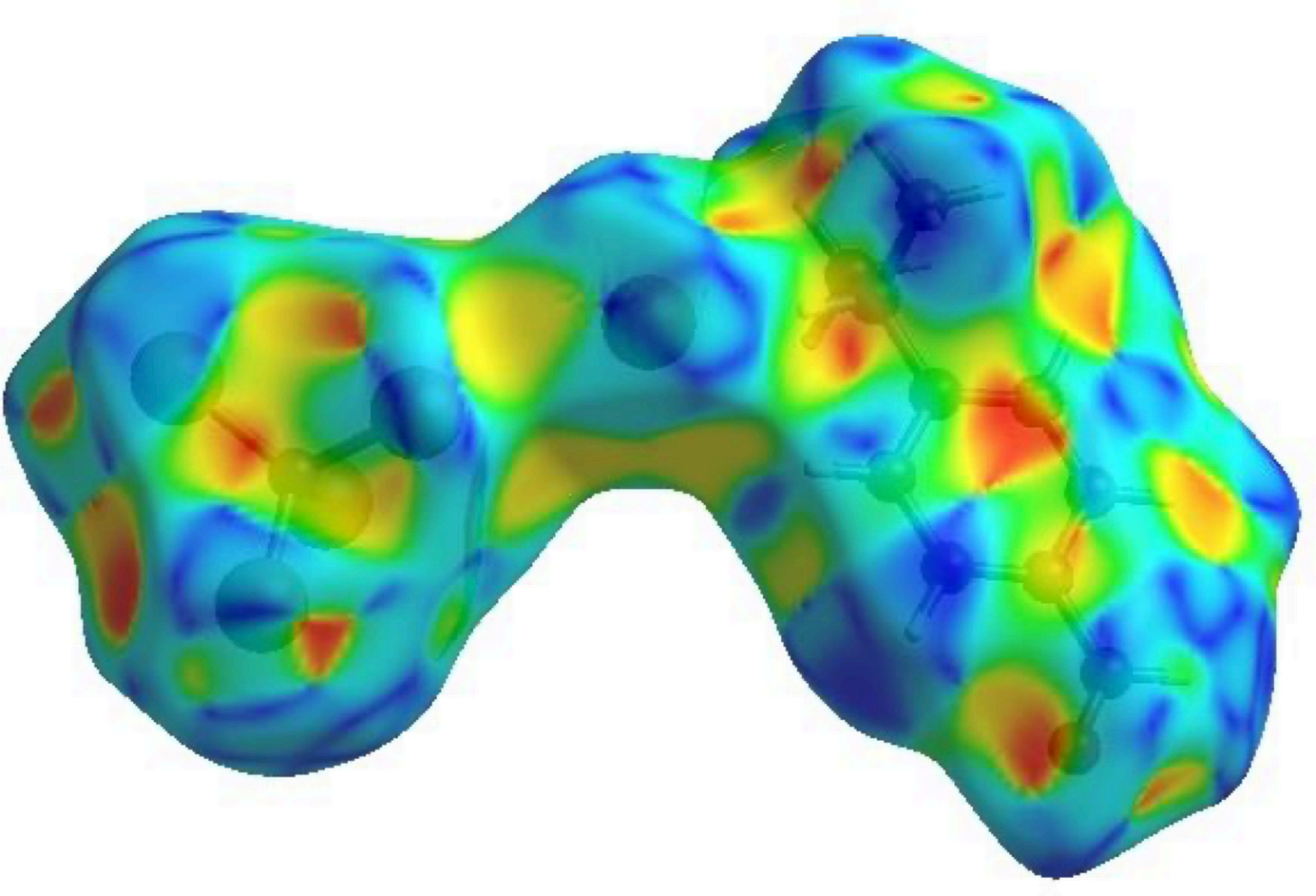
Hirshfeld surface of the title compound plotted over shape-index.

**Figure 5:**
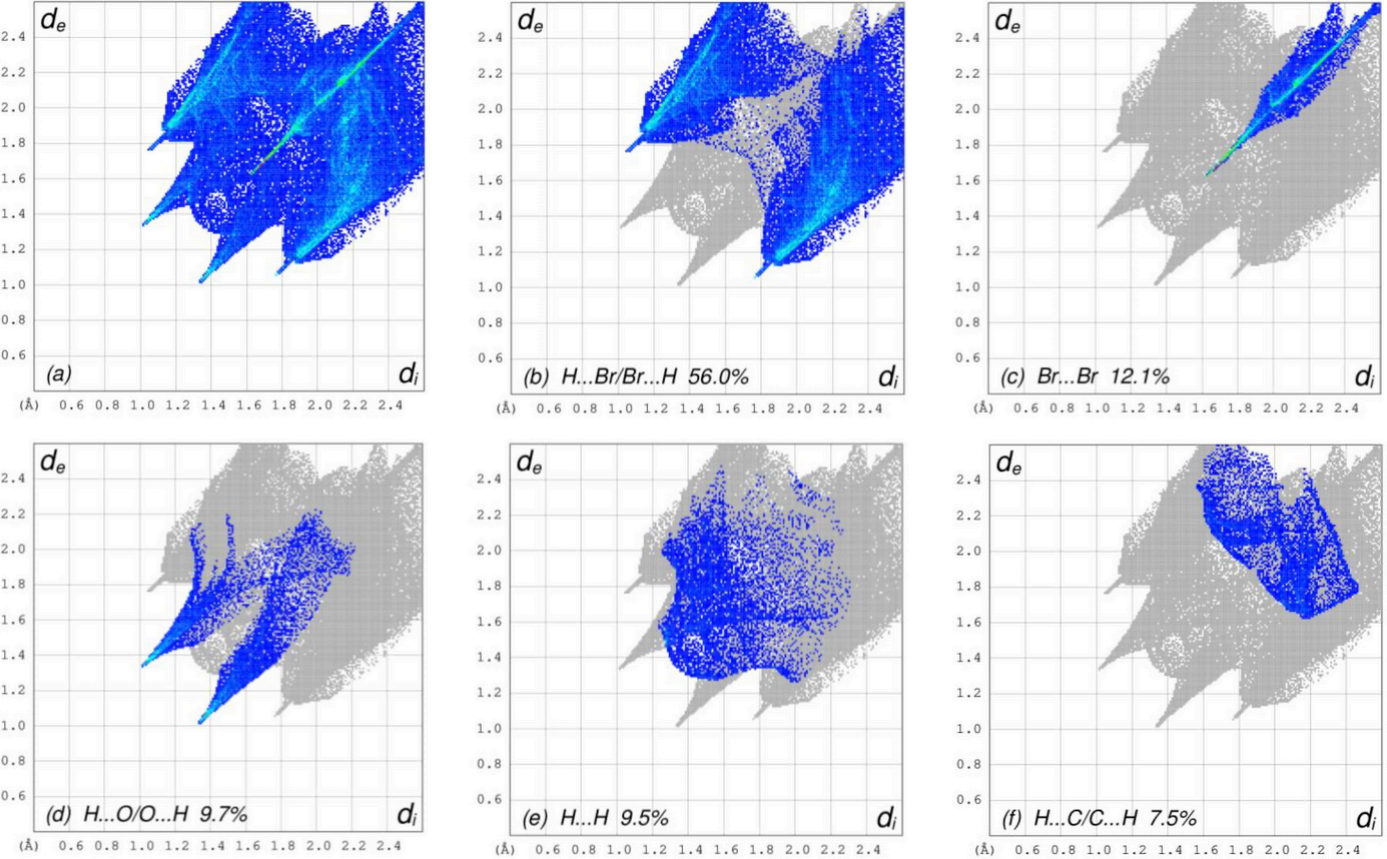
Two-dimensional HS-fingerprint plots showing, (*a*) all inter­actions, and those delineated into (*b*)H⋯Br/Br⋯H, (*c*) Br⋯Br, (*d*) H⋯O/O⋯H, (*e*) H⋯H, (*f*) H⋯C/C⋯H inter­actions. The *d*_i_ and *d*_e_ values are the closest inter­nal and external distances (in Å) from given points on the Hirshfeld surface.

**Figure 6:**
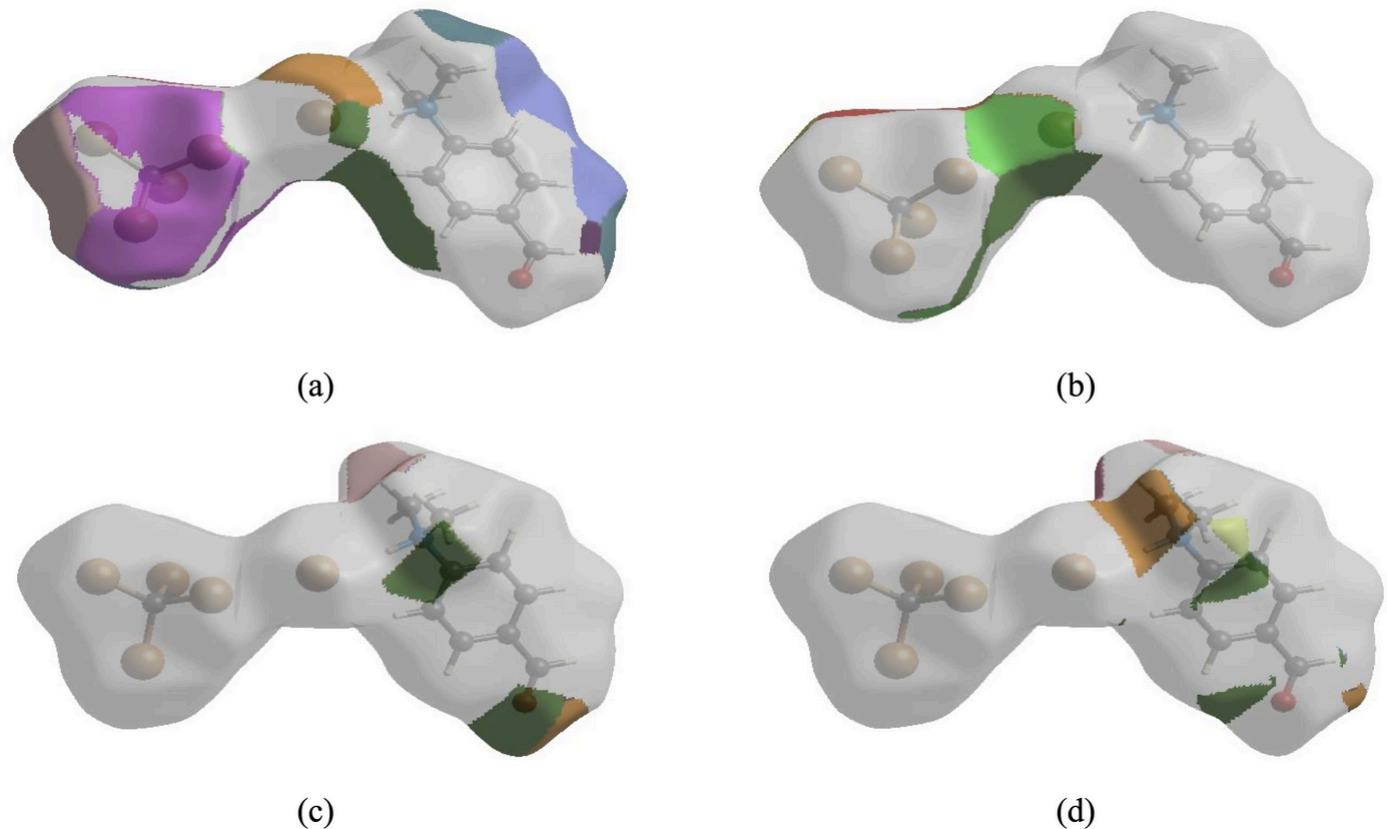
The Hirshfeld surface representations plotted as fragment patches for (*a*) H⋯Br/Br⋯H, (*b*) Br⋯Br, (*c*) H⋯O/O⋯H and (*d*) H⋯H inter­actions.

For visualizing the inter­molecular inter­actions in (I) a Hirshfeld surface (HS) analysis (Hirshfeld, 1977 ▸; Spackman & Jayatilaka, 2009 ▸) was carried out using *Crystal Explorer 17.5* (Spackman *et al.*, 2021 ▸). In the HS plotted over *d*_norm_ (Fig. 3 ▸), the white regions indicate contacts with distances equal to the sum of van der Waals radii, while the red and blue colours indicate distances shorter (in close contact) or longer (distant contact) than the sum of the van der Waals radii, respectively (Venkatesan *et al.*, 2016 ▸), where the bright-red spots indicate their roles as the respective donors and/or acceptors. There are no π–π stacking inter­actions between aromatic rings in the packing of (I). Unusually, this is in spite of the presence of juxtaposed red/blue triangular regions in the HS plotted over shape-index (Fig. 4 ▸). There are also no C—H⋯π close contacts. According to the two-dimensional fingerprint plots (McKinnon *et al.*, 2007 ▸), the inter­molecular H⋯Br/Br⋯H, Br⋯Br, H⋯O/O⋯H, H⋯H and H⋯C/C⋯H contacts make the most abundant contributions to the HS of 56%, 12.1%, 9.7%, 9.5% and 7.5% respectively (Table 2 ▸, Fig. 5 ▸). All other contact types contribute <5% to the surface. The nearest neighbour coordination environment of a mol­ecule can be determined from the colour patches on the HS based on how close to other mol­ecules they are. These are plotted onto the HS for the H⋯Br/Br⋯H, Br⋯Br, H⋯O/O⋯H and H⋯H inter­actions in Fig. 6 ▸, showing that van der Waals inter­actions and hydrogen bonding play the major roles in the crystal packing (Hathwar *et al.*, 2015 ▸).

## 5. Database survey

A substructure search of the Cambridge Structural Database [CSD Version 5.46 (November 2024); Groom, *et al.*, 2016 ▸] using the 4-formyl-*N*,*N*-di­methyl­anilinium moiety was carried out, and 49 similar compounds were found. Of these compounds, seven are structurally related. These include: *p*-di­methyl­amino-benzaldehyde hydro­bromide, C_9_H_12_NOBr (CSD refcode MABZAL10; Dattagupta & Saha, 1973 ▸), 4-formyl-*N*,*N*-di­methyl­anilinium 4-methyl­benzene­sulfonate monohydrate, C_9_H_12_NO^+^·C_7_H_7_O_3_S^−^·H_2_O (CSD refcode QAFROH; Jin *et al.*, 2016*a* ▸), ammonium 4-formyl-*N*,*N*-di­methyl­anilinium naphthalene-1,5-di­sulfonate ammonia, C_9_H_12_NO^+^·C_10_H_6_O_6_S_2_^2−^·H_4_N^+^·H_3_N (CSD refcode SUYYUI; Jin *et al.*, 2016*b* ▸), 4-formyl-*N*,*N*-di­methyl­anilinium tetra­fluoro­borate, C_9_H_12_NO^+^·BF_4_^−^ (CSD refcode VOJMEO; Froschauer *et al.*, 2013 ▸) and 4-formyl-*N*,*N*-di­methyl­anilinium 2,4,6-tri­nitro­phenolate, C_9_H_12_NO^+^·C_6_H_2_N_3_O_7_^−^ (CSD refcodes VUWLIJ: Thakuria *et al.*, 2007 ▸; VUWLIJ01: Jin *et al.*, 2016**a* ▸;* VUWLIJ02: Prasad, 2016 ▸). It is worth mentioning that the last three entries report different colours for the crystals (brown, colourless and metallic dark red).

## 6. Synthesis and crystallization

4-(Di­methyl­amino)­benzaldehyde (5 mmol) and tetra­bromo­methane (5 mmol) were dissolved in 25 ml of CHBr_3_, and left for slow evaporation. Orange crystals (suitable for X-ray analysis) of the product started to form after 1 d at room temperature; they were then filtered off and dried in air. Yield 59% (based on tetra­bromo­methane), orange powder soluble in methanol, ethanol and DMSO. Analysis calculated for C_10_H_12_Br_5_NO (*M*_r_ = 561.73): C, 21.38; H, 2.15; N, 2.49. Found: C, 21.36; H, 2.13; N, 2.47. ^1^H NMR (DMSO-*d*^6^), δ: 5.13 (–NHMe_2_), 9.67 (CHO), 7.70 and 7.68 (2H Ar), 6.80 and 6.78 (2H Ar), 3.05 (6H, 2CH_3_). ^13^C NMR (DMSO-*d*^6^), −29.2 (CBr_4_), 43.6 (2CH_3_), 111.2 (2C_Ar_), 124.4 (CCHO), 133.6 (2C_Ar_), 155.1 (CNMe_2_), 190.0 (C=O).

## 7. Refinement

Crystal data, data collection and structure refinement details are summarized in Table 3 ▸. The N- and C-bound hydrogen-atom positions were calculated geometrically at distances of 0.85 Å (for NH), 0.95 Å (for CH) and 0.98 Å (for CH_3_) and refined using a riding model by applying the constraint *U*_iso_ = *kU*_eq_ (C, N), where *k* = 1.2 for NH and CH hydrogen atoms and *k* = 1.5 for CH_3_ hydrogen atoms.

## Supplementary Material

Crystal structure: contains datablock(s) I. DOI: 10.1107/S2056989025006929/pk2717sup1.cif

Structure factors: contains datablock(s) I. DOI: 10.1107/S2056989025006929/pk2717Isup2.hkl

Supporting information file. DOI: 10.1107/S2056989025006929/pk2717Isup3.cml

CCDC reference: 2477918

Additional supporting information:  crystallographic information; 3D view; checkCIF report

## Figures and Tables

**Table 1:** Hydrogen-bond geometry (Å, °)

*D*—H⋯*A*	*D*—H	H⋯*A*	*D*⋯*A*	*D*—H⋯*A*
N1—H1*N*⋯Br4	0.85	2.37	3.221 (4)	175
C6—H6*A*⋯Br4	0.95	2.91	3.698 (4)	141
C7—H7*A*⋯O1^iii^	0.98	2.52	3.424 (5)	153
C7—H7*B*⋯O1^iv^	0.98	2.47	3.390 (5)	157

Symmetry codes: (iii) 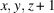; (iv) 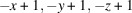.

**Table 2:** Selected interatomic distances (Å)

Br3⋯Br4^i^	3.3403 (8)	H7*B*⋯O1^iv^	2.47
H6*A*⋯Br3^ii^	2.95	C2⋯H7*C*	2.83
O1⋯H5*A*	2.57	C7⋯H2*A*	2.96
H7*A*⋯O1^iii^	2.52	H1*N*⋯H6*A*	2.22

Symmetry codes: (i) 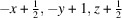; (ii) 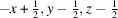; (iii) 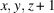; (iv) 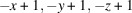.

**Table 3:** Experimental details

Crystal data	
Chemical formula	C_9_H_12_NO^+^·Br^−^·CBr_4_
*M*_r_	561.76
Crystal system, space group	Orthorhombic, *Pnma*
Temperature (K)	150
*a*, *b*, *c* (Å)	21.1900 (7), 7.4114 (2), 10.2297 (4)
*V* (Å^3^)	1606.55 (9)
*Z*	4
Radiation type	Mo *K*α
μ (mm^−1^)	12.49
Crystal size (mm)	0.32 × 0.18 × 0.12

Data collection	
Diffractometer	Bruker APEXII CCD
Absorption correction	Multi-scan (*SADABS*; Krause *et al.*, 2015 ▸)
*T*_min_, *T*_max_	0.086, 0.254
No. of measured, independent and observed [*I* > 2σ(*I*)] reflections	9073, 1649, 1462
*R*_int_	0.027
(sin θ/λ)_max_ (Å^−1^)	0.611

Refinement	
*R*[*F*^2^ > 2σ(*F*^2^)], *wR*(*F*^2^), *S*	0.026, 0.064, 1.06
No. of reflections	1649
No. of parameters	98
No. of restraints	6
H-atom treatment	H-atom parameters constrained
Δρ_max_, Δρ_min_ (e Å^−3^)	0.79, −1.21

Computer programs: *APEX3* and *SAINT* (Bruker, 2014 ▸), *SHELXT2019/1* (Sheldrick, 2015*a* ▸), *SHELXL2019/1* (Sheldrick, 2015*b* ▸) and *SHELXTL* (Sheldrick, 2008 ▸).

## supplementary crystallographic information

### 4-Formyl-N,N-dimethylanilinium bromide–tetrabromomethane (1/1) Crystal data

**Table:**

C_9_H_12_NO^+^·Br^−^·CBr_4_	*D*_x_ = 2.323 Mg m^−^^3^
*M**_r_* = 561.76	Mo *K*α radiation, λ = 0.71073 Å
Orthorhombic, *P**n**m**a*	Cell parameters from 4744 reflections
*a* = 21.1900 (7) Å	θ = 2.8–25.7°
*b* = 7.4114 (2) Å	µ = 12.49 mm^−^^1^
*c* = 10.2297 (4) Å	*T* = 150 K
*V* = 1606.55 (9) Å^3^	Plate, orange
*Z* = 4	0.32 × 0.18 × 0.12 mm
*F*(000) = 1048	

### 4-Formyl-N,N-dimethylanilinium bromide–tetrabromomethane (1/1) Data collection

**Table:**

Bruker APEXII CCD diffractometer	1462 reflections with *I* > 2σ(*I*)
φ and ω scans	*R*_int_ = 0.027
Absorption correction: multi-scan (SADABS; Krause *et al.*, 2015)	θ_max_ = 25.7°, θ_min_ = 2.8°
*T*_min_ = 0.086, *T*_max_ = 0.254	*h* = −25→25
9073 measured reflections	*k* = −9→8
1649 independent reflections	*l* = −11→12

### 4-Formyl-N,N-dimethylanilinium bromide–tetrabromomethane (1/1) Refinement

**Table:**

Refinement on *F*^2^	Primary atom site location: structure-invariant direct methods
Least-squares matrix: full	Secondary atom site location: difference Fourier map
*R*[*F*^2^ > 2σ(*F*^2^)] = 0.026	Hydrogen site location: mixed
*wR*(*F*^2^) = 0.064	H-atom parameters constrained
*S* = 1.06	*w* = 1/[σ^2^(*F*_o_^2^) + (0.0299*P*)^2^ + 4.3541*P*] where *P* = (*F*_o_^2^ + 2*F*_c_^2^)/3
1649 reflections	(Δ/σ)_max_ = 0.001
98 parameters	Δρ_max_ = 0.79 e Å^−^^3^
6 restraints	Δρ_min_ = −1.21 e Å^−^^3^

### 4-Formyl-N,N-dimethylanilinium bromide–tetrabromomethane (1/1) Special details

**Table:**

Geometry. All esds (except the esd in the dihedral angle between two l.s. planes) are estimated using the full covariance matrix. The cell esds are taken into account individually in the estimation of esds in distances, angles and torsion angles; correlations between esds in cell parameters are only used when they are defined by crystal symmetry. An approximate (isotropic)treatment of cell esds is used for estimating esds involving l.s. planes.

### 4-Formyl-N,N-dimethylanilinium bromide–tetrabromomethane (1/1) Fractional atomic coordinates and isotropic or equivalent isotropic displacement parameters (Å^2^)

**Table:**

	*x*	*y*	*z*	*U*_iso_*/*U*_eq_	
Br1	0.27771 (2)	0.53801 (5)	0.91970 (4)	0.02322 (12)	
Br2	0.16582 (3)	0.750000	0.78589 (6)	0.03084 (16)	
Br3	0.17920 (2)	0.750000	1.09645 (6)	0.02633 (15)	
Br4	0.39332 (2)	0.250000	0.88637 (6)	0.02293 (15)	
O1	0.53538 (19)	0.250000	0.1926 (4)	0.0295 (9)	
N1	0.54209 (18)	0.250000	0.8217 (4)	0.0160 (9)	
H1N	0.502359	0.250000	0.833231	0.019*	
C1	0.55221 (17)	0.250000	0.6801 (5)	0.0158 (10)	
C2	0.61354 (18)	0.250000	0.6293 (5)	0.0219 (11)	
H2A	0.649120	0.250000	0.685839	0.026*	
C3	0.6211 (2)	0.250000	0.4967 (5)	0.0244 (12)	
H3A	0.662508	0.250000	0.460938	0.029*	
C4	0.5690 (2)	0.250000	0.4127 (5)	0.0195 (11)	
C5	0.5087 (2)	0.250000	0.4645 (4)	0.0247 (12)	
H5A	0.473132	0.250000	0.407806	0.030*	
C6	0.4998 (2)	0.250000	0.6000 (4)	0.0227 (12)	
H6A	0.458558	0.250000	0.636234	0.027*	
C7	0.56696 (17)	0.4161 (5)	0.8872 (3)	0.0201 (8)	
H7A	0.556697	0.412102	0.980521	0.030*	
H7B	0.547604	0.523040	0.847635	0.030*	
H7C	0.612859	0.421721	0.876162	0.030*	
C8	0.5776 (3)	0.250000	0.2704 (5)	0.0268 (13)	
H8A	0.619581	0.250000	0.237724	0.032*	
C9	0.2245 (2)	0.750000	0.9317 (5)	0.0193 (11)	

### 4-Formyl-N,N-dimethylanilinium bromide–tetrabromomethane (1/1) Atomic displacement parameters (Å^2^)

**Table:**

	*U*^11^	*U*^22^	*U*^33^	*U*^12^	*U*^13^	*U*^23^
Br1	0.01931 (18)	0.0224 (2)	0.0280 (2)	0.00397 (14)	0.00079 (15)	0.00030 (15)
Br2	0.0245 (3)	0.0342 (3)	0.0338 (4)	0.000	−0.0135 (2)	0.000
Br3	0.0197 (3)	0.0320 (3)	0.0273 (3)	0.000	0.0072 (2)	0.000
Br4	0.0177 (2)	0.0244 (3)	0.0266 (3)	0.000	0.0002 (2)	0.000
O1	0.039 (2)	0.038 (2)	0.012 (2)	0.000	−0.0011 (18)	0.000
N1	0.0140 (19)	0.024 (2)	0.010 (2)	0.000	−0.0006 (16)	0.000
C1	0.015 (2)	0.020 (2)	0.012 (3)	0.000	0.0019 (19)	0.000
C2	0.013 (2)	0.036 (3)	0.016 (3)	0.000	−0.002 (2)	0.000
C3	0.013 (2)	0.038 (3)	0.022 (3)	0.000	0.006 (2)	0.000
C4	0.021 (2)	0.025 (3)	0.013 (3)	0.000	0.002 (2)	0.000
C5	0.022 (3)	0.042 (3)	0.011 (3)	0.000	−0.003 (2)	0.000
C6	0.014 (2)	0.041 (3)	0.013 (3)	0.000	0.000 (2)	0.000
C7	0.0262 (17)	0.0209 (19)	0.0132 (19)	−0.0021 (15)	−0.0019 (15)	−0.0029 (15)
C8	0.027 (3)	0.036 (3)	0.018 (3)	0.000	0.008 (2)	0.000
C9	0.013 (2)	0.022 (3)	0.022 (3)	0.000	0.001 (2)	0.000

### 4-Formyl-N,N-dimethylanilinium bromide–tetrabromomethane (1/1) Geometric parameters (Å, º)

**Table:**

Br1—C9	1.937 (3)	C3—C4	1.3998 (10)
Br2—C9	1.942 (5)	C3—H3A	0.9500
Br3—C9	1.940 (5)	C4—C5	1.384 (7)
O1—C8	1.198 (7)	C4—C8	1.467 (7)
N1—C1	1.464 (6)	C5—C6	1.3995 (10)
N1—C7^i^	1.497 (4)	C5—H5A	0.9500
N1—C7	1.497 (4)	C6—H6A	0.9500
N1—H1N	0.8501	C7—H7A	0.9800
C1—C6	1.379 (6)	C7—H7B	0.9800
C1—C2	1.3997 (10)	C7—H7C	0.9800
C2—C3	1.366 (7)	C8—H8A	0.9500
C2—H2A	0.9500		
			
Br3···Br4^ii^	3.3403 (8)	H7B···O1^v^	2.47
H6A···Br3^iii^	2.95	C2···H7C	2.83
O1···H5A	2.57	C7···H2A	2.96
H7A···O1^iv^	2.52	H1N···H6A	2.22
			
C1—N1—C7^i^	113.0 (2)	C6—C5—H5A	119.9
C1—N1—C7	113.0 (2)	C1—C6—C5	118.8 (4)
C7^i^—N1—C7	110.6 (4)	C1—C6—H6A	120.6
C1—N1—H1N	106.4	C5—C6—H6A	120.6
C7^i^—N1—H1N	106.7	N1—C7—H7A	109.5
C7—N1—H1N	106.6	N1—C7—H7B	109.5
C6—C1—C2	121.8 (4)	H7A—C7—H7B	109.5
C6—C1—N1	118.0 (3)	N1—C7—H7C	109.5
C2—C1—N1	120.2 (4)	H7A—C7—H7C	109.5
C3—C2—C1	118.6 (4)	H7B—C7—H7C	109.5
C3—C2—H2A	120.7	O1—C8—C4	124.5 (5)
C1—C2—H2A	120.7	O1—C8—H8A	117.7
C2—C3—C4	121.1 (4)	C4—C8—H8A	117.7
C2—C3—H3A	119.4	Br1—C9—Br1^vi^	108.4 (2)
C4—C3—H3A	119.4	Br1—C9—Br3	110.07 (18)
C5—C4—C3	119.6 (4)	Br1^vi^—C9—Br3	110.07 (18)
C5—C4—C8	119.6 (4)	Br1—C9—Br2	108.90 (18)
C3—C4—C8	120.8 (5)	Br1^vi^—C9—Br2	108.90 (18)
C4—C5—C6	120.2 (4)	Br3—C9—Br2	110.5 (2)
C4—C5—H5A	119.9		
			
C7^i^—N1—C1—C6	116.7 (3)	C2—C3—C4—C8	180.000 (1)
C7—N1—C1—C6	−116.7 (3)	C3—C4—C5—C6	0.000 (1)
C7^i^—N1—C1—C2	−63.3 (3)	C8—C4—C5—C6	180.000 (1)
C7—N1—C1—C2	63.3 (3)	C2—C1—C6—C5	0.000 (1)
C6—C1—C2—C3	0.000 (1)	N1—C1—C6—C5	180.000 (1)
N1—C1—C2—C3	180.000 (1)	C4—C5—C6—C1	0.000 (1)
C1—C2—C3—C4	0.000 (1)	C5—C4—C8—O1	0.000 (1)
C2—C3—C4—C5	0.000 (1)	C3—C4—C8—O1	180.000 (1)

Symmetry codes: (i) *x*, −*y*+1/2, *z*; (ii) −*x*+1/2, −*y*+1, *z*+1/2; (iii) −*x*+1/2, *y*−1/2, *z*−1/2; (iv) *x*, *y*, *z*+1; (v) −*x*+1, −*y*+1, −*z*+1; (vi) *x*, −*y*+3/2, *z*.

### 4-Formyl-N,N-dimethylanilinium bromide–tetrabromomethane (1/1) Hydrogen-bond geometry (Å, º)

**Table:**

*D*—H···*A*	*D*—H	H···*A*	*D*···*A*	*D*—H···*A*
N1—H1*N*···Br4	0.85	2.37	3.221 (4)	175
C6—H6*A*···Br4	0.95	2.91	3.698 (4)	141
C7—H7*A*···O1^iv^	0.98	2.52	3.424 (5)	153
C7—H7*B*···O1^v^	0.98	2.47	3.390 (5)	157

Symmetry codes: (iv) *x*, *y*, *z*+1; (v) −*x*+1, −*y*+1, −*z*+1.
